# Urbanisation is associated with reduced *Nosema* sp. infection, higher colony strength and higher richness of foraged pollen in honeybees

**DOI:** 10.1007/s13592-020-00758-1

**Published:** 2020-04-08

**Authors:** Ash E. Samuelson, Richard J. Gill, Ellouise Leadbeater

**Affiliations:** 1 School of Biological Sciences, Royal Holloway University of London, Egham, UK; 2 Department of Life Sciences, Imperial College London, Silwood Park Campus, Ascot, UK

**Keywords:** urbanisation, *Nosema* spp., *Varroa destructor*, pollen foraging, colony strength

## Abstract

Bees are vital pollinators, but are faced with numerous threats that include loss of floral resources and emerging parasites amongst others. Urbanisation is a rapidly expanding driver of land-use change that may interact with these two major threats to bees. Here we investigated effects of urbanisation on food store quality and colony health in honeybees (*Apis mellifera*) by sampling 51 hives in four different land-use categories: urban, suburban, rural open and rural wooded during two seasons (spring and autumn). We found positive effects of urban land use on colony strength and richness of stored pollen morphotypes, alongside lower late-season *Nosema* sp. infection in urban and suburban colonies. Our results reveal that honeybees exhibit lower colony performance in strength in rural areas, adding to the growing evidence that modern agricultural landscapes can constitute poor habitat for insect pollinators.

**Electronic supplementary material:**

The online version of this article (10.1007/s13592-020-00758-1) contains supplementary material, which is available to authorized users.

## Introduction

Honeybees (*Apis mellifera* L.) pollinate a significant proportion of the world’s crops and wild plants (Grünewald 2010). Therefore, reports of environmental threats to honeybees have generated concern regarding provision of pollination services, particularly as demand for pollination increases (Aizen and Harder 2009). Several threats to honeybee stocks have been implicated, including habitat loss and the associated lack of forage (Couvillon et al. 2014b; Alaux et al. 2017), parasites and disease (Brosi et al. 2017), and environmental contaminants such as pesticides (Henry et al. 2012; Wood and Goulson 2017).

While numbers of managed hives in the UK (Alton and Ratnieks 2013) and some parts of Europe (Potts et al. 2010; Breeze et al. 2014) have declined in recent decades, urban areas may be an exception due largely to an increase in popularity of urban beekeeping (Alton and Ratnieks 2013; Lorenz and Stark 2015). In London, the number of beekeepers rose from 464 to 1237 between 2008 and 2013 and the number of hives doubled to over 3500 (Alton and Ratnieks 2013), while in Berlin, the number of beekeepers increased by 53% and hives by 44% between 2006 and 2012 (Lorenz and Stark 2015). At the same time, urban areas are expanding, with a three-fold increase in global urban land cover estimated between 2000 and 2030 (Seto et al. 2012) and a predicted increase in global human urban population from 55 to 68% between 2018 and 2050 (United Nations 2018). Honeybees and other pollinators are therefore likely to come into increasing contact with cities in the future. However, research to date has generated mixed results regarding the effect of urbanisation on honeybee colony success (Sponsler and Johnson 2015; Garbuzov et al. 2015b; Lecocq et al. 2015; Youngsteadt et al. 2015).

Urban areas may offer more abundant and consistent forage than intensive agricultural areas in the form of flowering plants in gardens and parks (Goddard et al. 2010; Samuelson et al. 2018, 2019). Pollen is an important food source for a honeybee colony, providing protein, lipids and micronutrients essential for colony development (Keller et al. 2005). However, most research on the effects of land use on honeybee nutrition focusses on nectar (e.g. Lecocq et al. 2015). Because the nutritional quality of pollen diet varies widely depending on the contribution of different plant species (Keller et al. 2005), land use is likely to have a strong effect on pollen diet quality (Donkersley et al. 2014). Urban areas may provide a diverse range of pollen sources (Garbuzov and Ratnieks 2014), which may have implications for colony health (Di Pasquale et al. 2013; Smart et al. 2016; Dolezal and Toth 2018), but to our knowledge, no study to date has demonstrated whether urbanisation affects the diversity of pollen collected by honeybees. Urbanisation also interacts with parasite and disease stressors (Dolezal and Toth 2018), with initial evidence indicating that some honeybee (Youngsteadt et al. 2015) and bumblebee (Goulson et al. 2012; Theodorou et al. 2016) diseases may be more prevalent in urban areas. This may be mediated by higher hive densities (Alton and Ratnieks 2013; Brosi et al. 2017), resource patchiness (Youngsteadt et al. 2015), temperature differences (Gago et al. 2013) and differences in beekeeper experience and practices in urban and rural areas (Alton and Ratnieks 2013).

Here we investigate a set of colony-level measures in an extensive network of 51 honeybee hives located across a gradient of urbanisation in South East England at two time points during the foraging season. To investigate the association between urbanisation and parasitisation, we measured *Nosema* sp. prevalence, a common microsporidian gut parasite of the honeybee (Fries et al. 2013), and infestation by the *Varroa destructor* mite, arguably the greatest current parasite threat to honeybee populations (Genersch 2010; Brosi et al. 2017). To investigate effects on foraging, we analysed the composition and morphotype diversity of pollen collected by bees in different land-use types.

## Materials and methods

### Site selection

Of the initial 123 beekeeper applications to participate in the experiment, we selected 51 study apiaries located across a gradient of urbanisation in South East England with the aim to maximise spatial independence and land-use type representativeness while also minimising collinearity of covariates. Apiaries using hive types other than National, Commercial, Langstroth and WBC hives were excluded from the study, as were commercial beekeepers (which made up a small proportion of beekeeper applications, and for whom beekeeping practices may differ from hobby beekeepers). Preliminary data exploration showed collinearity between apiary size (number of hives) and land use, and beekeeper experience and land use, with larger apiaries and more experienced beekeepers in rural areas (see Supplementary Material). Furthermore, several sites were non-independent (< 6000 m apart; foraging ranges likely to overlap; Samuelson et al. 2019). To eliminate both issues, the following site selection protocol was carried out. Where two or more sites were less than 6000 m apart, only one site was chosen to be in the study based on the following objectives (in order): (1) maximising number of sites, (2) balanced representation of land-use types, (3) minimising collinearity (e.g. rural apiaries with few hives were preferred to those with many) and (4) maximising geographical spread.

### Land-use classification

**Figure 1.**
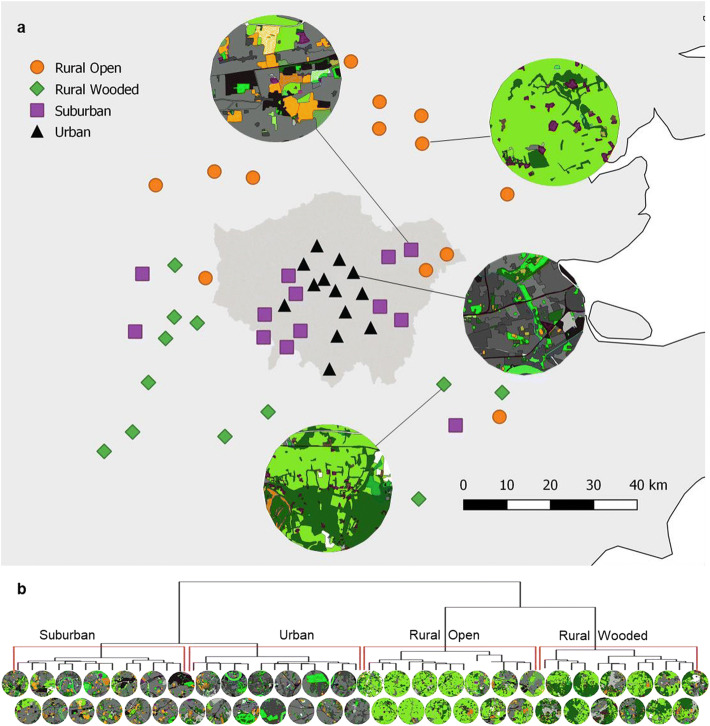
**a** Location of 51 apiary sites in SE England. The Greater London region is identified by dark grey shading, and inset circles show GIS land-use mapping for a representative site from each of the four land-use types (urban, suburban, rural open and rural wooded). Land-use map colours indicate 29 land classes; in summary, grey colours represent urban land classes (darker with increasing building density); green colours represent vegetated land including arable, pasture, woodland and urban parkland; orange colours represent sport and recreational open spaces; and blue colours represent water. **b** Cluster analysis (Ward’s method) of land-use types of 51 sites located in SE England; branch terminals show land-use maps of individual sites generated from GIS classification.

We classified land use at a radius of 3000 m around each site (radius based on the 99th percentile of waggle dance communicated distances from a separate study; Samuelson, Schuerch and Leadbeater, unpublished data). Classification was carried out in QGIS v2.16 following methods outlined in Samuelson and Leadbeater (2018). Briefly, land-use patches were defined by drawing polygons in QGIS over a satellite imagery baselayer (Bing Maps) and categorised visually to one of 29 land-use classes. Each land-use class was then coded to one of seven categories (impervious surface, domestic infrastructure, tree cover, gardens, open land, road and agricultural land) and the total area of each category within each site calculated. A PCA was performed to reduce the dimensionality of the land-use variables, and cluster analysis (Ward’s method with a minimum cluster size of five; Bunce et al. 1996; Hall and Arnberg 2002; Owen et al. 2006) was performed on the first two principle components (defined as urban-ness and openness), which in combination captured 82.2% of the variation. Four clear clusters emerged (Figure 1), comprising a group characterised by high urban-ness scores with mid-level openness scores (urban, *n* = 13 colonies), a group with high urban-ness and high openness scores (suburban, *n* = 13 colonies), a group with low urban-ness and high openness (rural open, *n* = 13 colonies) and a group with low urban-ness and low openness (rural wooded, *n* = 12 colonies). This grouping was used as a categorical land-use variable in all subsequent analyses.

### Sample collection

Two periods of sample collection were carried out: one in the early season (1–27 May 2015; hereafter “spring”) to coincide with the oilseed rape bloom (Free and Ferguson 1980) and one in the late season (18 August–14 September 2015; hereafter “autumn”) to coincide with late summer floral resource scarcity (Couvillon et al. 2014a). Sites were grouped into land-use types and a visit sequence constructed that equally distributed land-use types across the 4 weeks of the experiment with timings of visit within week depending on beekeeper availability, with a maximum of three apiaries visited each day. Each site was visited once in each period in approximately the same order. Sample collection was carried out between 10:00 and 17:00 on suitable days (> 12 °C, wind speed < 20 kmh). Samples were collected from one (queenright) hive per apiary, selected as the left-most hive from the apiary entrance. Site data for each apiary included GPS coordinates, number of hives in the apiary and number of colonies surviving the previous winter. Hive data included hive type (National, Commercial etc.) and disease treatment history since 2013. Colony strength (bee-covered surface) was estimated following standard methods by counting the number of sides of frames in which more than 50% of the surface area was covered with bees and recording the size (deep/shallow) and type (National, Commercial etc.) of frame to allow later calculation of covered surface area (Delaplane et al. 2013).

Thirty returning foragers were collected at the hive entrance to test for *Nosema* sp. (foragers are more likely to harbour the parasite; Fries et al. 2013). Taking 300 nurse bees per colony, the level of *Varroa destructor* mite infestation was assessed using the icing sugar shake method (Macedo et al. 2002). A frame from the lowest brood box containing brood at all stages and freshly stored pollen was shaken into a washtub and the flying bees allowed to leave. A cup of c. 300 bees (100 ml) was collected and tipped into a jar containing 1-tbsp icing sugar (Tate & Lyle, London, UK). This was rolled to ensure all bees were covered in sugar and left for 5 min in the shade, after which the icing sugar was shaken through the lid of the jar (size 8 hardware mesh) into a resealable plastic food bag. This was later dissolved in water, the number of mites counted three times and the mode taken.

We collected pollen samples from beebread as this has been shown to provide similar data to that derived from pollen traps and can be collected in a single hive visit (Dimou and Thrasyvoulou 2007). From the same frame as the *Varroa* samples, we followed the protocol of Tsvetkov et al. (2017) and used a spatula (width = 5 mm) to collect freshly stored pollen from thirty cells which was then placed in individual Eppendorf tubes per cell. Pollen cells were selected on the basis of freshness (powdery texture, no nectar seal). This pollen was likely to have been collected within 2 weeks prior to sampling (Vásquez and Olofsson 2009). All of the samples described above were placed immediately into dry ice and then into storage at – 80 °C within a maximum of 2 days.

### Pollen analysis and parasite screening

A small portion of each pollen sample (total = 2746 separate pollen samples) was mounted individually on a slide with water, basic fuchsin and glycerine jelly (Brunel Microscopes, Chippenham, UK) on a hotplate set to 80 °C. These were examined using a light microscope (Nikon Eclipse 50i) at × 400 magnification and each pollen morphotype was given a unique number, differentiated by established pollen morphological characteristics such as size, exine structure, shape and number of apertures. Because each pollen sample came from a single cell and the sample was scraped from the top layer of beebread within the cell, samples were typically homogenous or overwhelmingly dominated by a single type, and as such we recorded a single pollen type per sample rather than collecting within-sample quantitative data. We therefore consider each pollen sample to approximately represent a single foraging trip. Pollen types were identified to morphotypes to generate diversity and species composition data. Where possible, we additionally identified pollen types to family, genus or species (spring: 50% of morphotypes identified; autumn: 49%), on the basis of pollen morphology and colour using a combination of identification guides (Sawyer et al. 1981; Moore et al. 1991; Pollen-Wiki 2016; AutPal 2017). There is evidence to suggest that trees are an important pollen source for bees (Keller et al. 2005; Donkersley 2019), and the abundance of trees may differ between urban and rural areas. We therefore categorised identified pollen types as “woody” (trees and shrubs) or “non-woody” (herbs).

Pooled samples of 30 bees per colony were microscopically screened for *Nosema* sp*.* following Fries et al. (2013) and Human et al. (2013) to obtain information on colony-level *Nosema* sp. infection. We removed the abdomens of 30 frozen bees per colony and ground them with 30 ml distilled water. A 14-μl aliquot of the suspension was transferred to a haemocytometer, and the number of spores in five squares was counted. We did not identify the spores to species as *Nosema apis* and *Nosema ceranae* cannot be reliably differentiated microscopically. To obtain the *Nosema* sp. load for 30 bees, the following formula was applied:

$$ \mathrm{sample}\ \mathrm{volume}\ \left(\mathrm{ml}\right)\times \left(\frac{\mathrm{total}\ \mathrm{no}.\mathrm{counted}\ \mathrm{particles}\times \mathrm{dilution}\ \mathrm{factor}\ \left(\partial \right)}{\mathrm{area}\ \mathrm{of}\ \mathrm{squares}\ \mathrm{counted}\ \left({\mathrm{mm}}^2\right)\times \mathrm{chamber}\ \mathrm{depth}\ \left(\mathrm{mm}\right)}\right) $$

### Statistical analysis

Pollen species composition was analysed using PERMANOVA to investigate whether communities differed between land-use types in spring and autumn. For all other analyses, we followed an information theoretic approach to model selection (Grueber et al. 2011). We used an “all-subset” approach to build a comparison set that comprised (1) the basic null model containing only the constant and residual variance; (2) a full model containing combinations of the variables land use (urban, suburban, rural open or rural wooded), season (autumn or spring) and log-transformed apiary size (number of hives), their two-way interaction and colony strength (*Varroa* analysis only); and (3) all subsets of the full model. We selected the model with the lowest AICc as the best fitting model(s) (Johnson and Omland 2004) unless at least one alternative model was within two AICc units, in which case parameter estimates were based on conditional model averaging (Symonds and Moussalli 2011; Grueber et al. 2011). Where mixed models were appropriate, site was included as a random effect in the full model and all subsets. A single extreme observation whereby a single species (*Impatiens glandulifera*) made up all thirty pollen samples from one hive was removed from the analysis of “proportion of pollen from woody plants” because it undermined the assumptions of our models; including it did not change the outcome. Final models were validated graphically to assess fit and check that assumptions had been met (Zuur and Ieno 2011), and examined for spatial autocorrelation by using a Moran’s *I* test on the residuals and graphically assessing the spatial pattern of residuals. No evidence of spatial autocorrelation was found for any of the analyses.

Binomial GLMMs were performed to analyse the proportion of pollen collected from woody species. Pollen morphotype richness (for each hive) was analysed using GLMMs with Poisson error structure, and the Shannon diversity of pollen morphotypes was analysed with linear mixed models after scaling of the dependent variable. Colony strength (estimated bee-covered surface) was analysed using linear mixed models. Overwintering success, reported by beekeepers as a proportion of hives surviving the previous winter, was modelled using GLMs with a binomial error structure. *Nosema* infection and *Varroa* infestation were analysed using zero-altered Poisson hurdle models to deal with the zero-inflated dataset (*hurdle* function in R package *pscl*), where the response is modelled as two processes—a binomial process and a zero-truncated Poisson process (Zuur and Ieno 2011). Thus, the parameter estimates from the hurdle model provide information on both the binary probability of any infection (binomial process) and the number of spores/mites if infected (Poisson process). *Nosema* spore counts were cuberoot transformed prior to analysis. Final models containing categorical variables were rerun with each factor level coded as the baseline variable to investigate pairwise differences between factor levels (Te Grotenhuis and Thijs 2015; see Table S3). We carried out additional analyses to test the relationships between *Varroa* treatment, land use and *Varroa* infestation (see Supplementary Methods, Online Resource 1).

All analyses were conducted in R version 3.2.1 (R Core Team 2018) using packages *MuMIn* (Barton 2018), *lme4* (Bates et al. 2015), *pscl* (Zeileis et al. 2008), *vegan* (Oksanen et al. 2018), *betapart* (Baselga et al. 2018), *beeswarm* (Eklund 2016) and *Hmisc* (Harrell Jr and Dupont 2018).

## Results

### Colony health

**Figure 2.**
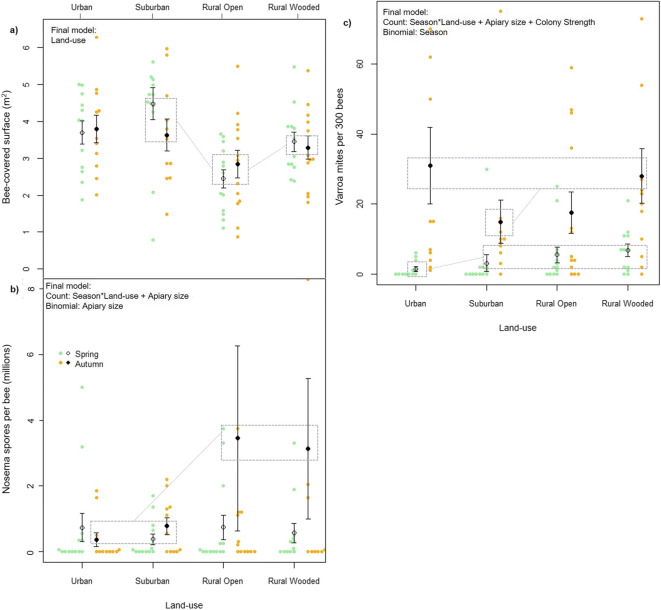
Means and standard errors for raw data for **a** colony strength, **b** *Varroa* mite count and **c** *Nosema* spore count across four land-use types in two periods, spring and autumn. Raw data are displayed as green (spring) and orange (autumn) points. Important significant pairwise differences with large effect sizes (see Table S3) are highlighted in grey boxes and variables included in the final model or model set are inset. LU land category.

Land category had a significant effect on colony strength (bee-covered surface), with the best model retaining only land category as a predictor (Figure 2a; ΔAICc to next best model = 3.05, Table S1a). Based on the pairwise interactions between land categories, we found model estimates of colony strength to be highest in suburban colonies followed by urban, rural wooded and lastly rural open (Table I(a)). Specifically, we found no statistical difference between the urban categories (urban parameter estimate with suburban as baseline [95% CIs]: − 0.345 [− 1.041 to 0.351]); however, when using suburban as a baseline intercept, both rural categories were significantly lower (rural open estimate with suburban as baseline: − 1.446 [− 2.121 to − 0.771]; rural open estimate with suburban as baseline: − 0.722 [− 1.411 to − 0.034]), and with urban as a baseline, we found rural open to be significantly lower (estimate: 1.101 [− 1.784 to − 0.481]). Only apiary size affected overwintering success, with a positive relationship between apiary size and success (Table S2b).

**Table I:** (a–e) Coefficients and 95% confidence intervals (CIs) for the optimal model or model sets (model averaged where applicable; see Table S1) for analyses where land use was included in the optimal model(s). Parameters highlighted in italics are considered important to the model (continuous variables) or significantly different from the baseline (categorical variables) based on 95% CIs not crossing zero. All analyses shown have urban as the baseline for land use and autumn as the baseline for season; for other baseline combinations see Table S3. (f) Results from PERMANOVA tests to analyse the effect of land use on pollen species composition in spring and autumn, and pairwise comparisons between land-use types in the spring

(a) Colony strength				
Parameters	Estimate	Std. error	95% CIs	
			Lower	Upper
(Intercept)	3.742	0.254	3.244	4.239
Land use (suburban)	0.345	0.355	− 0.351	1.041
*Land use* (*rural open*)	*− 1.101*	*0.349*	*− 1.784*	*− 0.418*
Land use (rural wooded)	− 0.377	0.355	− 1.073	0.319
(b) *Nosema*				
Parameters	Estimate	Std. error	95% CIs	
			Lower	Upper
Count process				
(Intercept)	4.499	0.057	4.387	4.611
Apiary size	− 0.015	0.018	− 0.050	0.021
Season (spring)	0.126	0.069	− 0.010	0.262
Land use (suburban)	0.109	0.065	− 0.019	0.237
*Land use* (*rural open*)	*0.397*	*0.062*	*0.275*	*0.520*
*Land use* (*rural wooded*)	*0.309*	*0.063*	*0.186*	*0.433*
*Land use* (*suburban*): *season* (*spring*)	*− 0.350*	*0.090*	*− 0.528*	*− 0.173*
*Land use* (*rural open*): *season* (*spring*)	*− 0.422*	*0.087*	*− 0.592*	*− 0.252*
*Land use* (*rural wooded*): *season* (*spring*)	*− 0.467*	*0.087*	*− 0.638*	*− 0.295*
Binomial process				
(Intercept)	− 0.251	0.495	− 1.222	0.720
Apiary size	0.440	0.283	− 0.115	0.994
(c) *Varroa*				
Parameters	Estimate	Std. error	95% CIs	
			Lower	Upper
Count process				
(Intercept)	3.809	0.069	3.674	3.944
*Apiary size*	*− 0.304*	*0.038*	*− 0.378*	*− 0.230*
*Season* (*spring*)	*− 2.295*	*0.266*	*− 2.816*	*− 1.773*
*Land use* (*suburban*)	*− 0.594*	*0.095*	*− 0.780*	*− 0.407*
*Land use* (*rural open*)	*− 0.187*	*0.087*	*− 0.358*	*− 0.017*
Land use (rural wooded)	0.074	0.078	− 0.079	0.227
*Land use* (*suburban*): *season* (*spring*)	*1.548*	*0.323*	*0.915*	*2.181*
*Land use* (*rural open*): *season* (*spring*)	*1.212*	*0.299*	*0.626*	*1.798*
*Land use* (*rural wooded*): *season* (*spring*)	*0.886*	*0.294*	*0.311*	*1.462*
Binomial process				
(Intercept)	2.128	0.473	1.201	3.055
*Season* (*spring*)	*− 1.757*	*0.555*	*− 2.845*	*− 0.669*
(d) Proportion woody pollen				
Parameters	Estimate	Std. error	95% CIs	
			Lower	Upper
(Intercept)	− 1.823	0.447	− 2.699	− 0.947
*Apiary size*	*− 0.366*	*0.184*	*− 0.727*	*− 0.005*
*Season* (*spring*)	*4.684*	*0.353*	*3.991*	*5.376*
Land use (suburban)	0.259	0.480	− 0.682	1.200
Land use (rural open)	− 0.653	0.491	− 1.615	0.309
Land use (rural wooded)	− 0.159	0.482	− 1.104	0.785
Land use (suburban): season (spring)	0.280	0.487	− 0.675	1.234
*Land use* (*rural open*): *season* (*spring*)	*− 1.030*	*0.450*	*− 1.912*	*− 0.148*
*Land use* (*rural wooded*): *season* (*spring*)	*− 1.014*	*0.449*	*− 1.895*	*− 0.134*
(e) Pollen species richness				
Parameters	Estimate	Std. error	95% CIs	
			Lower	Upper
(Intercept)	2.041	0.115	1.814	2.267
piary size	− 0.055	0.061	− 0.174	0.064
Season (spring)	− 0.102	0.101	− 0.299	0.095
Land use (suburban)	− 0.076	0.158	− 0.386	0.233
*Land use* (*rural open*)	*− 0.316*	*0.135*	*− 0.581*	*− 0.051*
Land use (rural wooded)	− 0.186	0.134	− 0.448	0.076
Land use (suburban): season (spring)	− 0.422	0.220	− 0.854	0.010
Land use (rural open): season (spring)	− 0.019	0.226	− 0.461	0.424
Land use (rural wooded): season (spring)	0.045	0.220	− 0.386	0.477
(f) Pollen species composition (PERMANOVA)				
Overall		*F*	*R*^2^	*p* value
*Spring*		*3.653*	*0.199*	*0.005*
Autumn		1.269	0.029	0.159
Pairs (spring)				
*Suburban vs urban*		*3.742*	*0.145*	*0.003*
*Suburban vs rural open*		*6.609*	*0.223*	*0.001*
*Suburban vs rural wooded*		*2.821*	*0.109*	*0.008*
*Urban vs rural open*		*4.578*	*0.179*	*0.001*
*Urban vs rural wooded*		*3.114*	*0.129*	*0.007*
Rural open vs rural wooded		1.464	0.062	0.190

Land category had a significant effect on *Nosema* spore count in hives, with highest prevalence in both rural categories in the autumn (Figure 2b). *Nosema* infection was analysed through hurdle models in which the count process models spore count, and the binomial process probability of infection. The binary probability of infection did not differ significantly between sites (only “Apiary size” was retained in the binomial process within the final model set; Table I). However, land category, season and their interaction were all retained within the count process of every model in the candidate set (Table I). In the autumn, both rural open and wooded sites showed significantly higher spore count than urban and suburban sites (rural open parameter estimate with urban as baseline: 0.397 [0.275 to 0.519]; rural wooded parameter estimate with urban as baseline: 0.310 [0.186 to 0.433]; Table S3e). Smaller but significant differences were found between land categories in the spring, with suburban displaying lower *Nosema* prevalence than urban and rural open (urban parameter estimate with suburban as baseline: 0.190 [0.072 to 0.307]; rural open estimate with suburban as baseline: 0.218 [0.102 to 0.334]; Table S3e, Figure 2b).

Land category had an effect on *Varroa* mite counts in those colonies that contained *Varroa* (count process of the hurdle model) but not on the probability that colonies would contain *Varroa* (binomial process). Land category, season and their interaction along with apiary size and colony strength were all retained in the count process but only season was retained in the binomial process of the best model (ΔAICc to next best model = 2.0; Table S1b). Unlike for *Nosema*, hives in all land categories showed higher *Varroa* mite counts in autumn than spring (autumn parameter estimate with urban and spring as baseline: 2.227 [1.705 to 2.750], Table S3d, Figure 2). There was no consistent effect of urbanisation, with the highest autumn counts in urban and rural wooded colonies (suburban parameter estimate with urban as baseline: − 0.604 [− 0.790 to − 0.417]; Table S3d, Figure 2c). In contrast, in spring, *Varroa* counts were lower in urban than all other land categories (suburban parameter estimate with urban as baseline: 0.841 [0.233 to 1.448]; rural open parameter estimate with urban as baseline: 1.123 [0.559 to 1.687]; rural wooded parameter estimate with urban as baseline: 0.942 [0.387 to 1.497]; Table S3d, Figure 2c).

### Pollen

#### Pollen richness and diversity

**Figure 3.**
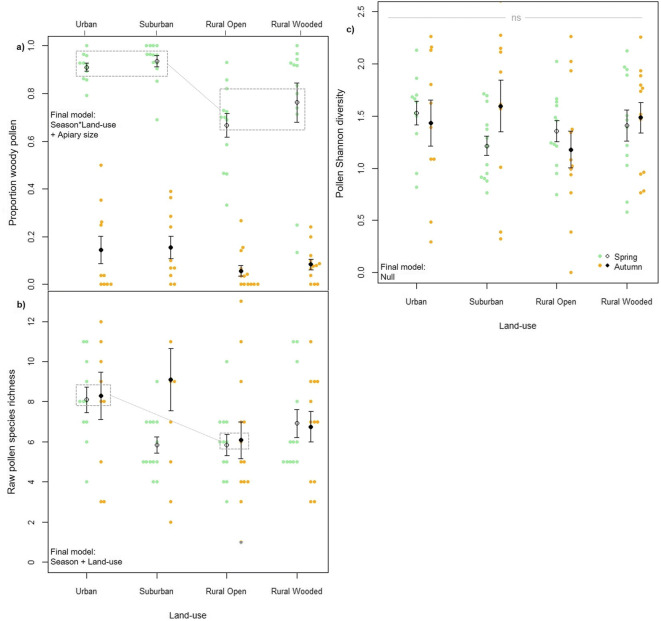
Means and standard errors for **a** proportion woody pollen, **b** pollen morphotype richness and **c** pollen Shannon diversity across four land-use types in two periods, spring and autumn. Raw data are displayed as green (spring) and orange (autumn) points. Important significant pairwise differences with large effect sizes (see Table S3) are highlighted in grey boxes and variables included in the final model or model set are inset.

Land category, season and their interaction along with apiary size all affected pollen morphotype richness (Table S1e), with higher richness in urban than rural open colonies (spring rural open parameter estimate with urban as baseline: − 0.320 [− 0.586 to − 0.054]; autumn rural open parameter estimate with urban as baseline: − 0.316 [− 0.581 to − 0.051]; Table S3b, Figure 3b). There was no effect on any of the measured variables on Shannon diversity (Figure 3c), with the null model showing the lowest AICc (ΔAICc to next best model: 3.26; Table S1f).

#### Morphotype composition

**Figure 4.**
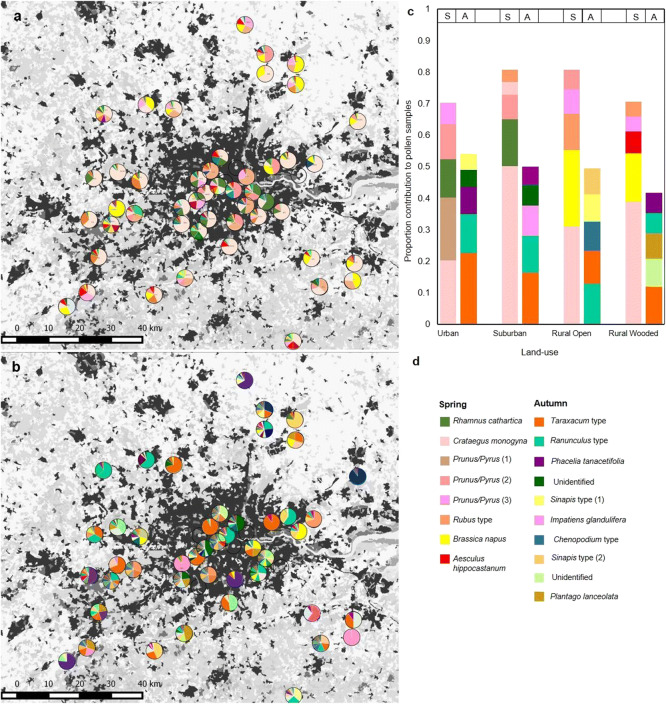
Contribution of different pollen types to pollen samples in each of 51 sites across SE England collected in **a** spring and **b** autumn. **c** Five most important pollen types (greatest contribution) in each of four land-use types in spring and autumn. **d** Colour legend for five most important pollen types in each land-use type. Additional pollen types and their colours are shown in the supplementary material (Online Resource 1).

**Figure 5.**
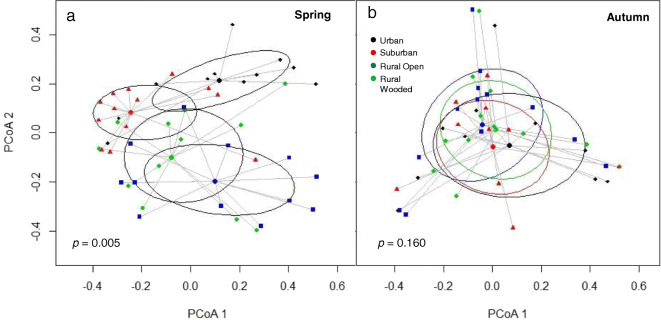
Principal coordinate analysis (PCoA) plots to visualise differences in pollen species composition in four land-use types in **a** spring and **b** autumn using Bray-Curtis distances (Beals 1984). Ellipses represent one standard deviation from the centroid and *p* values from PERMANOVA analyses are inset. Points represent individual sites: urban (black diamonds), suburban (red triangles), rural wooded (green diamonds), rural open (blue squares).

In spring, the five most common pollen types in each land category made up on average 75.7 ± SE 3.0% of samples across land categories compared to 48.8 ± 2.6% in autumn (Figure 4c). Spring-collected pollen composition differed significantly between land categories (PERMANOVA, *df* = 3, *F* = 3.6, *p* = 0.005). Pollen morphotype composition did not significantly differ between rural wooded and rural open land categories, but composition in these land categories differed significantly from those in urban and suburban, which also differed from each other (Figure 5a; Table I(f)). In the autumn, collected pollen composition was not significantly affected by land category (PERMANOVA, *df* = 1, *F* = 1.2, *p* = 0.160; Figure 5b). These differences were reflected in the differences between land catgeories in proportion of pollen collected from woody plants. Land category, season and their interaction along with apiary size affected the proportion of pollen collected from woody plants (ΔAICc to next best model: 7.64; Figure 3a, Table S1d). A significantly greater proportion of pollen was collected from woody plants in spring (mean ± SE: 81.9 ± 2.9%) than in autumn (mean 10.5 ± 1.9%; autumn parameter estimate with spring and urban as baseline: 4.700 [4.004 to 5.395]; Table I(d), Table S3a). In spring, woody plants made up a greater proportion of collected pollen in urban and suburban than either rural land categories (rural open parameter estimate with suburban as baseline: − 2.220 [− 3.062 to − 1.379]; rural wooded parameter estimate with suburban as baseline: − 1.728 [− 2.591 to − 0.865]) while in autumn, there were no significant differences between land categories (Table I(d), Table S3a).

## Discussion

We found positive effects of urban land use on honeybee colony strength and the raw morphotype richness of collected pollen, alongside lower late-season counts for *Nosema* sp. infection in hives from urban and suburban sites. Colony strength was lower in rural open colonies than all other land-use types, a result that aligns with recent findings of lower bumblebee colony growth in colonies placed in agricultural sites compared to urbanised sites (Samuelson et al. 2018). This may be related to higher *Nosema* sp. infection in rural sites, which may have affected colony growth (Genersch 2010). Lower forage availability in rural sites may also have limited colony growth (Garbuzov et al. 2015b; Lecocq et al. 2015 although cf. Sponsler and Johnson 2015; Sponsler et al. 2017). Moreover, nutritional quality (protein content) of honeybee-collected pollen has been reported to be higher in built-up areas (Donkersley et al. 2017). The presence of pesticide residues in the foraging landscape also has the potential to affect colony growth in honeybees (Tsvetkov et al. 2017; Woodcock et al. 2017; although cf. Rundlöf et al. 2015) and has been shown to be higher in rural than urban areas (Botías et al. 2017; Nicholls et al. 2018 although cf. Johnson and Pettis 2014).

Our finding of higher autumn *Nosema sp.* spore counts in rural sites relative to urbanised sites contradicts the findings from a US study which showed higher *Nosema ceranae* loads in honeybee colonies (*n* = 39) in more urbanised areas (Youngsteadt et al. 2015). The higher loads in hives at rural sites in our study could reflect a greater prevalence of *Nosema* sp. in the environment. This is plausible given the use of commercial bumblebee colonies in agricultural environments which have the potential to lead to pathogen spillover to honeybees visiting the same plants (Graystock et al. 2013; Fürst et al. 2014), and that more concentrated resource patches in agricultural areas such as mass-flowering crops could lead to increased localised bee densities (Requier et al. 2015) leading to pathogen transmission hotspots, a phenomenon that may be exacerbated in landscapes with little semi-natural habitat or other floral resources (Piot et al. 2019).

*Varroa* mite counts were higher in autumn (mean 22.7 mites per 300 bees) than spring (mean 4.2), following typical patterns of build-up over the season (Rosenkranz et al. 2010), but did not follow a consistent pattern with land use. In autumn, *Varroa* counts were highest in urban and rural wooded colonies, a result not driven by *Varroa* treatment (see Supplementary Results, Online Resource 1). Colony strength and apiary size affected *Varroa* counts, with higher *Varroa* counts in larger colonies and smaller apiaries. Local hive density (not measured here) may be a potential driver behind differences in parasite prevalence between land-use types. Horizontal disease transmission through drifting, robbing or shared foraging resources (Fries and Camazine 2001) may be increased in areas with higher colony densities, as has been shown for *Paenibacillus larvae* (American foulbrood) (Lindström et al. 2008), an important honeybee disease. Hive densities may be higher in urban than rural areas due to the popularity of urban beekeeping and increased human population density (Alton and Ratnieks 2013; Lorenz and Stark 2015), although varied apicultural and agricultural practices drive local and temporal differences in rural hive densities (Lindström et al. 2008). As such, future research should examine the relationship between differences in local honeybee colony density between urban and rural areas and disease prevalence.

Identification of the pollen collected by colonies highlights important forage plants in urban and rural areas and suggests that a richer variety of floral resources are available in cities, with raw morphotype richness (but not Shannon diversity) higher in urban than rural open colonies. This may be due to agricultural intensification reducing the available pollen sources in farmland areas (Lecocq et al. 2015) in contrast to the highly diverse array of exotic and native flowering plants available in urban gardens (Garbuzov and Ratnieks 2014). Pollen diet richness has implications for honeybee health (Dolezal and Toth 2018), supporting immune function and glucose oxidase activity (an enzyme involved in food store sterilisation; Alaux et al. 2010), reducing disease mortality (Di Pasquale et al. 2013) and promoting acquisition of beneficial gut microorganisms (Anderson et al. 2013; Corby-Harris et al. 2016). However, high diet richness may also indicate a lack of availability of high-volume resources such as mass-flowering crops, precluding bees from specialising on a small number of abundant species, as may be possible in agricultural areas (Rollin et al. 2013). Research indicates that honeybees in agricultural areas visit mass-flowering crops when they are available and then switch to semi-natural patches with less dense and more varied floral resources when the bloom is over (González-Varo and Vilà 2017; Samuelson et al. 2019), suggesting that a rich diet may be evidence that high-volume resources are unavailable.

Spring pollen morphotype composition was strongly differentiated by land-use type, with distinct urban, suburban and rural (open + wooded) groups, while in autumn, there was no difference in composition across land use, possibly because important pollen sources in autumn samples consisted of generalist plants such as *Taraxacum* (dandelion) and *Ranunculus* (buttercup) which are common weeds in both agricultural and urban landscapes (Sterry 2008; Hicks et al. 2016). In contrast, important spring sources were more likely to consist of agricultural or urban specialists, such as *Rhamnus cathartica* in urban (buckthorn, a common hedge plant; Kurylo and Endress 2007) and *Brassica napus* in rural areas (oilseed rape, a widespread agricultural crop; Garbuzov et al. 2015a). This is intensified by the fact that the five most important species made up a larger contribution (mean across land-use types 76%) in spring than in autumn (mean 49%), suggesting that colonies focussed collection more on these common plant sources in the spring than in the autumn. Spring pollen sources were dominated by woody plants (reflecting results from previous research; Keller et al. 2005; Donkersley 2019), and urban and suburban colonies collected a significantly higher proportion of woody pollen than rural colonies. This reliance on pollen from trees and shrubs in the spring, when demand for protein is high while colonies are building up brood (Keller et al. 2005), highlights the importance of urban trees in otherwise high-density built-up areas (Macivor et al. 2014). In rural areas, *B. napus* contributed a substantial proportion of spring-collected pollen. However, no colony fed exclusively on *B. napus* when it was available, with the highest proportion of *B.napus* pollen at 53% and the mean (excluding colonies containing no *B. napus* pollen) at 22.6 ± 4% (reflecting the 14% average found in a study by Garbuzov et al. 2015a). This has implications for colony exposure to pesticides. Many studies calculate exposure assuming exclusive foraging on *B. napus* while it is in bloom (Whitehorn et al. 2012; Henry et al. 2012); our findings, like those of Garbuzov et al. (2015a), suggest that this would result in an overestimation of pesticide exposure. However, it is important to note that nectar collection may rely more heavily on mass-flowering crops than pollen (Samuelson et al. 2019; Requier et al. 2015).

Our findings demonstrate largely positive effects of urbanisation on honeybee colony success, complementing a growing body of evidence suggesting that urban areas may also support populations of wild pollinators (Baldock et al. 2015; Hall et al. 2016; Samuelson et al. 2018). While this has positive implications for the recent rise in urban beekeeping (Lorenz and Stark 2015), it is important to note that our findings also serve to highlight the poor suitability of modern agricultural habitats for honeybees and many other pollinator taxa. Consequently, conservation efforts should focus on improving these habitats for pollinators.

## Electronic supplementary material

ESM 1. (DOCX 123 kb)

ESM 2. (PDF 584 kb)
