# Supplementary material for: Urbanisation is associated with reduced Nosema sp. infection, higher colony strength and higher richness of foraged pollen in honeybees
Source: Apidologie. 2020 Apr 8;51(5):746–62. doi: 10.1007/s13592-020-00758-1 (PMC7584562; doi:10.1007/s13592-020-00758-1)
Supplement: Supplementary file 1 — (DOCX 123 kb) [file 13592_2020_758_MOESM1_ESM.docx]

# Urbanisation is associated with reduced *Nosema* sp. infection, higher colony strength and higher richness of foraged pollen in honeybees

# Online Resource 1

Ash E. Samuelson*, Richard J. Gill & Ellouise Leadbeater

School of Biological Sciences, Royal Holloway University of London, Egham, United Kingdom

*Corresponding author: [ash.samuelson.2014@live.rhul.ac.uk](mailto:ash.samuelson.2014@live.rhul.ac.uk)

**Supplementary Methods**

We carried out additional analyses to test the relationships between *Varroa* treatment, land-use and *Varroa* infestation. To test the effect of recent *Varroa* treatment on *Varroa* infestation, we reran the analysis for both seasons separately including the categorical covariate *Varroa treatment* as this was collinear with season. We retained the variable *land-use* as a chi-square test suggested this was not collinear with treatment. We grouped treatments carried out in the previous 6 months into “none”, “physical” (including shook swarm and icing sugar), “thymol”, “oxalic acid”, “formic acid” and “pyrethroid”, and within each season excluded treatment groups with <3 observations, resulting in the groups “none” and “oxalic” in spring and “thymol”, “formic acid” and “none” in autumn. We carried out an additional analysis to investigate whether land-use affected beekeepers’ decision to use *Varroa* treatment. We ran binomial GLMMs on *Varroa* treatment as a binary response with data from winter and summer treatments over the last three years for each site, with site as a random effect.

**Supplementary Results and Discussion**

Analysis of the effect of recent (≤ 6 months) *Varroa* treatment on mite count (separate models for each season due to seasonal differences in treatment practices) found that treatment in addition to land-use had an effect on *Varroa* infestation (Tables S1i & S1j). In spring, colonies that had been treated with oxalic acid had lower mite counts than those that had not been treated in the last six months, while in autumn colonies that had been treated with formic acid had fewer mites than those treated with thymol, which in turn had fewer than those not treated at all (Fig S2; Tables S2d & S2e). A formal analysis of the association between land-use and treatment practice found that there was no effect of land-use on the likelihood to use *Varroa* treatment; in other words beekeepers in one land-use type were no less likely to treat their hives than those in another (Tables S1h & S2c), suggesting that differences in mite counts between land-use types do not reflect differing treatment between land-use types. Apiary size also had a small negative association with *Varroa* infestation (independently of land-use as the apiary size of our sites was balanced across land-use types), meaning that larger apiaries had slightly lower mite counts.

Overall, the unbalanced pollen composition with a high contribution of common plants (overall mean: 62%) reflect findings from previous research, with the identity of important species also matching those found in other studies (review: Keller, Fluri and Imdorf, 2005).

**Supplementary Figures**

**Figure S1.** List of pollen types and colour legend for Figs 1a and b. Where it was not possible to visually identify pollen types to species/genus/family, morphotypes were given a unique number.

| **SPRING** |  | **AUTUMN** |
| --- | --- | --- |
| *1* |  | *Taraxacum* type |
| *Crataegus monogyna* |  | *Plantago lanceolata* type |
| *Prunus/Pyrus*(A) |  | *3* |
| *Prunus/Pyrus*(B) |  | *Sinapis* type (A) |
| *Brassica napus* |  | *Sinapis* type (B) |
| *Vicia faba* |  | *Lonicera* |
| *Aesculus hippocastaneum* |  | *6* |
| *6* |  | *7* |
| *7* |  | *Ranunculus* type (A) |
| *Rubus* type |  | *Ranunculus* type (B) |
| *Brunnera macrophylla* |  | *9* |
| *9* |  | *10* |
| *Prunus/Pyrus*(C) |  | *11* |
| *Prunus/Pyrus*(D) |  | *Rosaceae* (A) |
| *11* |  | *13* |
| *12* |  | *Curcubita pepo* |
| *13* |  | *Ericaceae* |
| *14* |  | *Anthemis* type (A) |
| *Ilex aquifolium* |  | *17* |
| *16* |  | *18* |
| *Iridaceae*(A) |  | *Rosaceae* (B) |
| *Cornus* |  | *Passiflora cereulea* |
| *19* |  | *21* |
| *20* |  | *23* |
| *21* |  | *Echinops sphaerocephalus* |
| *Ranunculus* type |  | *Mentha* type |
| *Linum catharticum* |  | *26* |
| *24* |  | *27* |
| *25* |  | *Buddleia* type (A) |
| *25.1* |  | *Buddleia* type (B) |
| *27* |  | *29* |
| *28* |  | *30* |
| *29* |  | *Anthemis* type (B) |
| *30* |  | *32* |
| *Taraxacum* type |  | *33* |
| *Ericaceae* |  | *34* |
| *33* |  | *Calystegia sepium* |
| *34* |  | *36* |
| *35* |  | *37* |
| *36* |  | *Eucalpytus* |
| *Mentha* type |  | *Anthemis* type (C) |
| *Lonicera* |  | *Centaurea nigra* |
| *39* |  | *41* |
| *40* |  | *Veronica* type |
| *41* |  | *Salvia(A)* |
| *Iridaceae*(B) |  | *Filipendula ulmaria* |
| *Echium* |  | *45* |
| *44* |  | *46* |
| Compositae type |  | *Impatiens glandulifera* |
| Malvaceae type |  | *48* |
| *47* |  | *Gladiolus* type |
| *48* |  | *50* |
| *Phacelia tanacetifolia* |  | *51* |
|  |  | *52* |
|  |  | *53* |
|  |  | *Phacelia tanacetifolia* |
|  |  | *Zea mays* |
|  |  | *Umbelliferaceae* (A) |
|  |  | *Umbelliferaceae* (B) |
|  |  | *Geranium/Pelargonium* |
|  |  | *Salvia*(B) |
|  |  | *61* |
|  |  | *Epilobium parviflorum* |
|  |  | *Borago officianalis* |
|  |  | *Chenopodium type* |
|  |  | *Veronica serpyllifolia* |
|  |  | *Iridaceae* type |
|  |  | *Oenothera biennis* |
|  |  | *68* |
|  |  | *69* |
|  |  | Compositae type |
|  |  | *Iridaceae* |
|  |  | *72* |
|  |  | *Asparagus* |
|  |  | *Begonia* |
|  |  | *Vicia faba* |
|  |  | *76* |
|  |  | *77* |
|  |  | *78* |
|  |  | *80* |
|  |  | *81* |
|  |  | *82* |
|  |  | *83* |

**Figure S2.** Mean (± SE) *Varroa* mite count for colonies treated with different *Varroa* treatments in the last six months. a) In spring colonies were either treated with no treatment or oxalic acid (sublimation or trickle). b) In autumn colonies were treated either with formic acid, thymol or no treatment. A small number of colonies were treated with a pyrethroid (n=1) or a physical method e.g. icing sugar or shook swarm (n=3); these were excluded from the analysis.**
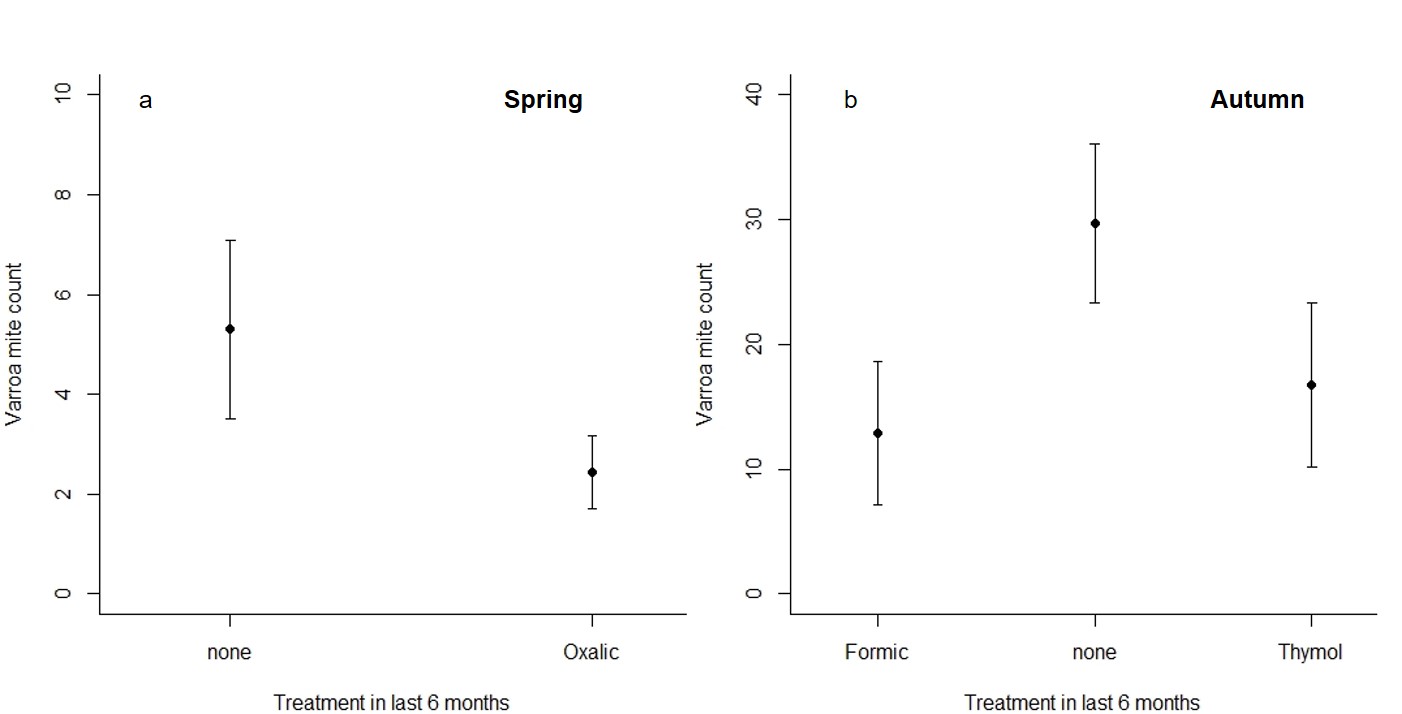
**

**Figure S3.** Data exploration of the initial 123 apiaries that applied to participate in the experiment showed collinearity between a) beekeeper experience (years) and land-use, and b) apiary size (number of hives) and land-use, with larger apiaries and more experienced beekeepers in rural areas, A site selection protocol was carried out to minimise this collinearity (see Main Text). Formal land classification was only carried out on the final 51 sites, with data exploration employing an initial visual classification to three broad land-use types: Urban, Suburban and Rural.


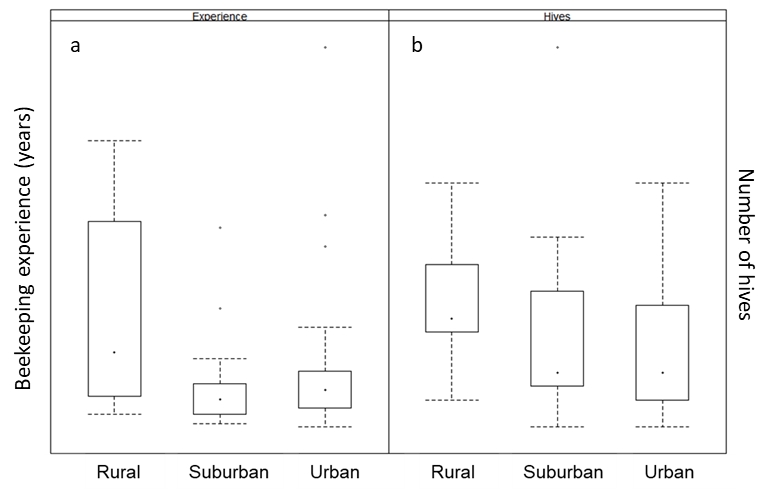


**Supplementary Tables**

See Appendix 2
