# Supplementary material for: Urbanisation is associated with reduced Nosema sp. infection, higher colony strength and higher richness of foraged pollen in honeybees
Source: Apidologie. 2020 Apr 8;51(5):746–62. doi: 10.1007/s13592-020-00758-1 (PMC7584562; doi:10.1007/s13592-020-00758-1)
Supplement: Supplementary file 2 — (PDF 584 kb) [file 13592_2020_758_MOESM2_ESM.pdf]

Supplementary Tables for Samuelson et al. Urbanisation is associated with reduced *Nosema* sp. infection, larger colonies and more species-rich pollen forage for honeybees

Authors: Ash E. Samuelson*, Richard J. Gill & Ellouise Leadbeater
*Royal Holloway, University of London; ash.samuelson.2014@live.rhul.ac.uk
Submitted to Apidologie

Table S1. Tables of candidate models using a full-subset information theoretic approach. In all cases, the basic model included the constant and the residual variance, with all other models containing the basic model plus the indicated covariates. Models are presented in order of ΔAICc from the best model alongside their respective Akaike weights (w_i); only the first four candidate models are shown. The best sets of models which were averaged to obtain model averaged estimates (models <2 ΔAICc from the model with the lowest AICc) are highlighted in bold.

	df	AICc	ΔAICc	w_i	Land-use	Season	Land-use: Season	Apiary Size
a) Colony strength								
**Model 7**	**6**	**325.7**	**0**	**0.638**	+			
Model 6	7	328.7	3.05	0.138	+	+		
Model 3	7	329.3	3.67	0.102	+			+
Model 5	10	329.8	4.18	0.079	+	+	+	

	df	AICc	ΔAICc	w_i	Count process: Land-use	Season	Land-use: Season	Apiary Size	Colony Strength	Binomial process: Land-use	Season	Land-use: Season	Apiary Size	Colony Strength
b) *Varroa*														
**Model 28**	**12**	**1482.5**	**0**	**0.48**	+	+	+	+	+		+			
Model 78	13	1484.5	2	0.18	+	+	+	+	+		+			+
Model 27	13	1484.9	2.37	0.15	+	+	+	+	+		+		+	
Model 26	15	1486.8	4.26	0.06	+	+	+	+	+	+	+			

c) *Nosema*	df	AICc	ΔAICc	w_i	Count process: Land-use	Season	Land-use: Season	Apiary Size	Binomial process: Land-use	Season	Land-use: Season	Apiary Size
**Model 55**	**10**	**1823**	**0**	**0.261**	+	+	+					+
**Model 5**	**9**	**1823**	**0.02**	**0.259**	+	+	+					
**Model 30**	**10**	**1824.8**	**1.85**	**0.103**	+	+	+	+				
**Model 80**	**11**	**1824.9**	**1.89**	**0.101**	+	+	+	+				+

	df	AICc	ΔAICc	w_i	Land-use	Season	Land-use: Season	Apiary Size
d) Proportion woody pollen								
**Model 6**	**10**	**504.5**	**0**	**0.697**	+	+	+	+
**Model 1**	**9**	**506.4**	**1.85**	**0.276**	+	+	+	
Model 7	7	512.2	7.64	0.015	+	+		+
Model 2	6	512.7	8.19	0.012	+	+		
e) Pollen species richness								
**Basic model**	**2**	**464.9**	**0**	**0.177**				
**Model 3**	**3**	**465**	**0.1**	**0.168**		+		
**Model 2**	**6**	**465.3**	**0.4**	**0.145**	+	+		
**Model 4**	**5**	**465.3**	**0.48**	**0.139**	+			
f) Pollen Shannon diversity								
**Basic model**	**3**	**271**	**0**	**0.689**				
Model 9	4	274.3	3.26	0.135				+
Model 3	4	274.5	3.47	0.121		+		
Model 7	5	277.8	6.75	0.024		+		+

	df	AICc	ΔAICc	w_i	Land-use	Apiary Size	Land-use: Apiary Size
g) Overwintering success							
**Model 4**	**2**	**81.6**	**0**	**0.661**		**+**	
**Basic model**	**1**	**84.5**	**2.89**	**0.156**			
Model 2	5	85.4	3.79	0.099	+	+	
Model 1	8	86.5	4.94	0.056	+	+	+
h) Effect of land-use on V*arroa* treatment use							
**Basic model**	**2**	**281.5**	**0**	**0.584**			
**Model 3**	**3**	**282.5**	**0.97**	**0.36**		+	
Model 2	5	287.1	5.64	0.035	+		
Model 1	6	288.2	6.7	0.021	+	+	

					Count process			Binomial process		
	df	AICc	ΔAICc	w_i	Land-use	Recent *Varroa* treatment	Apiary Size	Land-use	Recent *Varroa* treatment	Apiary Size
i) *Varroa* treatment effect on *Varroa* mite count (spring)										
**Model 20**	**7**	**233.1**	**0**	**0.417**	**+**	**+**	**+**			
Model 19	10	235.5	2.38	0.127	+	+	+	+		
Model 36	8	235.6	2.44	0.123	+	+	+			+
Model 18	8	235.9	2.77	0.104	+	+	+		+	
j) *Varroa* treatment effect on *Varroa* mite count (autumn)										
**Model 20**	**8**	**970.2**	**0**	**0.671**	**+**	**+**	**+**			
Model 36	9	973.2	3.01	0.149	+	+	+			+
Model 18	10	973.8	3.59	0.111	+	+	+		+	
Model 19	11	975.9	5.69	0.039	+	+	+	+		

Table S2. a) Results from PERMANOVA tests to analyse the effect of land-use on pollen species composition in spring and autumn, and pairwise comparisons between land-use types in the autumn. b-e) Coefficients and 95% confidence intervals (CIs) for the optimal model or model sets (model averaged where applicable; see Table S1) for analyses of b) overwintering success, c) land-use effect on *Varroa* treatment and d & e) *Varroa* treatment effect on mite count. Parameters highlighted in bold are considered important to the model (continuous variables) or significantly different from the baseline (categorical variables) based on 95% CIs.

a) Pollen species composition (PERMANOVA)

Overall	F	R^2	P value
**Spring**	**3.653**	**0.199**	**0.005**
Autumn	1.269	0.029	0.159
Pairs (autumn)			
Suburban vs Urban	0.575	0.031	0.962
Suburban vs Rural Open	0.896	0.041	0.591
Suburban vs Rural Wooded	0.969	0.046	0.508
Urban vs Rural Open	0.681	0.031	0.861
Urban vs Rural Wooded	0.918	0.044	0.570
Rural Open vs Rural Wooded	1.075	0.045	0.346

b) Overwintering success

Parameters	Estimate	Std. Error	95% CIs	
			Lower	Upper
(Intercept)	0.860	0.514	-0.148	1.867
**Apiary Size**	**0.618**	**0.278**	**0.073**	**1.163**

c) Land-use effect on *Varroa* treatment

Parameters	Estimate	Std. Error	95% CIs	
			Lower	Upper
(Intercept)	0.487	0.338	-0.177	1.150
Apiary Size	0.251	0.241	-0.222	0.724

d) *Varroa* treatment effect on *Varroa* mite count (spring)

Parameters	Estimate	Std. Error	95% CIs	
			Lower	Upper
Count process				
(Intercept)	2.075	0.365	1.360	2.791
**Apiary Size**	**-0.322**	**0.121**	**-0.559**	**-0.084**
Land-use (Suburban)	-0.939	0.628	-2.169	0.291
Land-use (Rural Open)	0.633	0.347	-0.047	1.313
**Land-use (Rural Wooded)**	**0.967**	**0.342**	**0.298**	**1.637**
**Recent *Varroa* treatment (Oxalic)**	**-0.620**	**0.182**	**-0.975**	**-0.264**
Binomial process				
(Intercept)	0.234	0.307	-0.368	0.836

e) *Varroa* treatment effect on *Varroa* mite count (autumn)				
Parameters	Estimate	Std. Error	95% CIs	
			Lower	Upper
Count process				
(Intercept)	3.121	0.106	2.913	3.329
**Apiary Size**	**-0.220**	**0.039**	**-0.297**	**-0.143**
**Land-use (Suburban)**	**-0.873**	**0.097**	**-1.062**	**-0.684**
**Land-use (Rural Open)**	**-0.505**	**0.092**	**-0.686**	**-0.324**
Land-use (Rural Wooded)	-0.143	0.079	-0.298	0.012
**Recent *Varroa* treatment (none)**	**1.073**	**0.097**	**0.883**	**1.262**
**Recent *Varroa* treatment (Thymol)**	**0.539**	**0.116**	**0.312**	**0.766**
Binomial process				
(Intercept)	2.079	0.474	1.150	3.009

Table S3. Coefficients and 95% confidence intervals (CIs) for the optimal model or model sets (model averaged where applicable; see Table 2 in main text) for all analyses where land-use was included in the optimal model. For each analysis the output is given with each combination of factor levels as the baseline to demonstrate pairwise comparisons. Parameters highlighted in bold are significantly different to the baseline based on 95% CIs.

a) Proportion woody pollen				
SEASON: AUTUMN / LAND-USE: URBAN				
Parameters	Estimate	Std. Error	95% CIs	
			Lower	Upper
(Intercept)	-1.693	0.417	-2.511	-0.875
**Apiary Size**	**-0.366**	**0.184**	**-0.727**	**-0.005**
**Season (Spring)**	**4.700**	**0.355**	**4.004**	**5.395**
Land-use (Suburban)	0.284	0.483	-0.663	1.231
Land-use (Rural Open)	-0.606	0.491	-1.568	0.357
Land-use (Rural Wooded)	-0.131	0.485	-1.080	0.819
Land-use (Suburban) : Season (Spring)	0.275	0.489	-0.684	1.233
**Land-use (Rural Open) : Season (Spring)**	**-1.056**	**0.451**	**-1.940**	**-0.172**
**Land-use (Rural Wooded) : Season (Spring)**	**-1.038**	**0.450**	**-1.921**	**-0.156**
SEASON: AUTUMN / LAND-USE: SUBURBAN				
Parameters	Estimate	Std. Error	95% CIs	
			Lower	Upper
(Intercept)	-1.409	0.429	-2.249	-0.568
**Apiary Size**	**-0.366**	**0.184**	**-0.727**	**-0.005**
**Season (Spring)**	**4.974**	**0.343**	**4.302**	**5.646**
Land-use (Urban)	-0.284	0.483	-1.231	0.663
Land-use (Rural Open)	-0.890	0.473	-1.816	0.036
Land-use (Rural Wooded)	-0.415	0.469	-1.334	0.504
Land-use (Urban) : Season (Spring)	-0.275	0.489	-1.233	0.684
**Land-use (Rural Open) : Season (Spring)**	**-1.330**	**0.440**	**-2.193**	**-0.467**
**Land-use (Rural Wooded) : Season (Spring)**	**-1.313**	**0.438**	**-2.171**	**-0.454**
SEASON: AUTUMN / LAND-USE: RURAL OPEN				
Parameters	Estimate	Std. Error	95% CIs	
			Lower	Upper
(Intercept)	-2.299	0.456	-3.193	-1.404
**Apiary Size**	**-0.366**	**0.184**	**-0.727**	**-0.005**
**Season (Spring)**	**3.644**	**0.278**	**3.099**	**4.188**
Land-use (Suburban)	0.890	0.473	-0.036	1.816
Land-use (Urban)	0.606	0.491	-0.357	1.568
Land-use (Rural Wooded)	0.475	0.473	-0.452	1.402
**Land-use (Suburban) : Season (Spring)**	**1.330**	**0.440**	**0.467**	**2.193**
**Land-use (Urban) : Season (Spring)**	**1.056**	**0.451**	**0.172**	**1.940**
Land-use (Rural Wooded) : Season (Spring)	0.017	0.392	-0.750	0.785

SEASON: AUTUMN / LAND-USE: RURAL WOODED				
Parameters	Estimate	Std. Error	95% CIs	
			Lower	Upper
(Intercept)	-1.824	0.433	-2.672	-0.976
**Apiary Size**	**-0.366**	**0.184**	**-0.727**	**-0.005**
**Season (Spring)**	**3.661**	**0.278**	**3.116**	**4.207**
Land-use (Rural Open)	-0.475	0.473	-1.402	0.452
Land-use (Suburban)	0.415	0.469	-0.504	1.334
Land-use (Urban)	0.131	0.485	-0.819	1.080
**Land-use (Rural Open) : Season (Spring)**	**-0.017**	**0.392**	**-0.785**	**0.751**
**Land-use (Suburban) : Season (Spring)**	**1.313**	**0.438**	**0.454**	**2.171**
Land-use (Urban) : Season (Spring)	1.038	0.450	0.156	1.921

SEASON: SPRING / LAND-USE: URBAN				
Parameters	Estimate	Std. Error	95% CIs	
			Lower	Upper
(Intercept)	3.007	0.422	2.180	3.833
**Apiary Size**	**-0.366**	**0.184**	**-0.727**	**-0.005**
**Season (Autumn)**	**-4.700**	**0.355**	**-5.395**	**-4.004**
**Land-use (Rural Wooded)**	**-1.169**	**0.434**	**-2.019**	**-0.319**
**Land-use (Rural Open)**	**-1.662**	**0.425**	**-2.494**	**-0.829**
Land-use (Suburban)	0.559	0.472	-0.366	1.483
**Land-use (Rural Wooded) : Season (Autumn)**	**1.038**	**0.450**	**0.156**	**1.921**
**Land-use (Rural Open) : Season (Autumn)**	**1.056**	**0.451**	**0.172**	**1.940**
Land-use (Suburban) : Season (Autumn)	-0.275	0.489	-1.233	0.684

SEASON: SPRING / LAND-USE: SUBURBAN				
Parameters	Estimate	Std. Error	95% CIs	
			Lower	Upper
(Intercept)	3.565	0.457	2.669	4.461
**Apiary Size**	**-0.366**	**0.184**	**-0.727**	**-0.005**
**Season (Autumn)**	**-4.974**	**0.343**	**-5.646**	**-4.302**
Land-use (Urban)	-0.559	0.472	-1.483	0.366
**Land-use (Rural Wooded)**	**-1.728**	**0.440**	**-2.591**	**-0.865**
**Land-use (Rural Open)**	**-2.220**	**0.430**	**-3.062**	**-1.378**
Land-use (Urban) : Season (Autumn)	0.275	0.489	-0.684	1.233
**Land-use (Rural Wooded) : Season (Autumn)**	**1.313**	**0.438**	**0.454**	**2.171**
**Land-use (Rural Open) : Season (Autumn)**	**1.330**	**0.440**	**0.467**	**2.193**

SEASON: SPRING / LAND-USE: RURAL OPEN				
Parameters	Estimate	Std. Error	95% CIs	
			Lower	Upper
(Intercept)	1.345	0.399	0.562	2.128
**Apiary Size**	**-0.366**	**0.184**	**-0.727**	**-0.005**
**Season (Autumn)**	**-3.644**	**0.278**	**-4.188**	**-3.099**
**Land-use (Suburban)**	**2.220**	**0.430**	**1.378**	**3.062**
**Land-use (Urban)**	**1.661**	**0.425**	**0.829**	**2.494**
Land-use (Rural Wooded)	0.492	0.390	-0.271	1.256
**Land-use (Suburban) : Season (Autumn)**	**-1.330**	**0.440**	**-2.193**	**-0.467**
**Land-use (Urban) : Season (Autumn)**	**-1.056**	**0.451**	**-1.940**	**-0.172**
Land-use (Rural Wooded) : Season (Autumn)	-0.017	0.392	-0.785	0.750

SEASON: SPRING / LAND-USE: RURAL WOODED				
Parameters	Estimate	Std. Error	95% CIs	
			Lower	Upper
(Intercept)	1.838	0.392	1.069	2.606
**Apiary Size**	**-0.366**	**0.184**	**-0.727**	**-0.005**
**Season (Autumn)**	**-3.661**	**0.278**	**-4.207**	**-3.116**
Land-use (Rural Open)	-0.492	0.390	-1.256	0.271
**Land-use (Suburban)**	**1.728**	**0.440**	**0.865**	**2.591**
**Land-use (Urban)**	**1.169**	**0.434**	**0.319**	**2.019**
Land-use (Rural Open) : Season (Autumn)	0.017	0.392	-0.750	0.785
**Land-use (Suburban) : Season (Autumn)**	**-1.313**	**0.438**	**-2.171**	**-0.454**
**Land-use (Urban) : Season (Autumn)**	**-1.038**	**0.450**	**-1.921**	**-0.156**

b) Pollen species richness				
SEASON: AUTUMN / LAND-USE: URBAN				
Parameters	Estimate	Std. Error	95% CIs	
			Lower	Upper
(Intercept)	2.041	0.115	1.814	2.267
Apiary Size	-0.055	0.061	-0.174	0.064
Season (Spring)	-0.102	0.101	-0.299	0.095
Land-use (Rural Wooded)	-0.186	0.134	-0.448	0.076
**Land-use (Rural Open)**	**-0.316**	**0.135**	**-0.581**	**-0.051**
Land-use (Suburban)	-0.076	0.158	-0.386	0.233
Land-use (Rural Wooded) : Season (Srping)	0.045	0.220	-0.386	0.477
Land-use (Rural Open) : Season (Spring)	-0.019	0.226	-0.461	0.424
Land-use (Suburban) : Season (Spring)	-0.422	0.220	-0.854	0.010
SEASON: AUTUMN / LAND-USE: SUBURBAN				
Parameters	Estimate	Std. Error	95% CIs	
			Lower	Upper
(Intercept)	2.010	0.111	1.792	2.228
Apiary Size	-0.055	0.061	-0.174	0.064
Season (Spring)	-0.166	0.151	-0.461	0.130
Land-use (Urban)	0.076	0.158	-0.233	0.386
Land-use (Rural Wooded)	-0.110	0.164	-0.431	0.211
Land-use (Rural Open)	-0.240	0.158	-0.550	0.070
Land-use (Urban) : Season (Spring)	0.422	0.220	-0.010	0.854
**Land-use (Rural Wooded) : Season (Spring)**	**0.468**	**0.221**	**0.034**	**0.901**
Land-use (Rural Open) : Season (Spring)	0.403	0.227	-0.042	0.848
SEASON: AUTUMN / LAND-USE: RURAL OPEN				
Parameters	Estimate	Std. Error	95% CIs	
			Lower	Upper
(Intercept)	1.912	0.132	1.654	2.171
Apiary Size	-0.055	0.061	-0.174	0.064
Season (Spring)	-0.105	0.101	-0.302	0.093
Land-use (Suburban)	0.240	0.158	-0.070	0.550
**Land-use (Urban)**	**0.316**	**0.135**	**0.051**	**0.581**
Land-use (Rural Wooded)	0.130	0.137	-0.138	0.398
Land-use (Suburban) : Season (Spring)	-0.404	0.227	-0.849	0.041
Land-use (Urban) : Season (Spring)	0.019	0.226	-0.424	0.461
Land-use (Rural Wooded) : Season (Spring)	0.064	0.227	-0.380	0.508

SEASON: AUTUMN / LAND-USE: RURAL WOODED				
Parameters	Estimate	Std. Error	95% CIs	
			Lower	Upper
(Intercept)	1.965	0.100	1.770	2.160
Apiary Size	-0.055	0.061	-0.174	0.064
Season (Spring)	-0.095	0.107	-0.306	0.115
Land-use (Rural Open)	-0.130	0.137	-0.398	0.138
Land-use (Suburban)	0.110	0.164	-0.211	0.431
Land-use (Urban)	0.186	0.134	-0.076	0.449
Land-use (Rural Open) : Season (Spring)	-0.064	0.227	-0.508	0.380
**Land-use (Suburban) : Season (Spring)**	**-0.468**	**0.221**	**-0.901**	**-0.034**
Land-use (Urban) : Season (Spring)	-0.046	0.220	-0.477	0.386

SEASON: SPRING / LAND-USE: URBAN				
Parameters	Estimate	Std. Error	95% CIs	
			Lower	Upper
(Intercept)	1.986	0.115	1.761	2.211
Apiary Size	-0.055	0.061	-0.174	0.064
Season (Autumn)	0.102	0.101	-0.095	0.299
Land-use (Rural Wooded)	-0.177	0.133	-0.438	0.083
**Land-use (Rural Open)**	**-0.320**	**0.136**	**-0.586**	**-0.054**
Land-use (Suburban)	-0.161	0.156	-0.468	0.146
Land-use (Rural Wooded) : Season (Autumn)	-0.045	0.220	-0.477	0.386
Land-use (Rural Open) : Season (Autumn)	0.018	0.226	-0.424	0.461
Land-use (Suburban) : Season (Autumn)	0.422	0.220	-0.010	0.854

SEASON: SPRING / LAND-USE: SUBURBAN				
Parameters	Estimate	Std. Error	95% CIs	
			Lower	Upper
(Intercept)	1.921	0.107	1.712	2.129
Apiary Size	-0.055	0.061	-0.174	0.064
Season (Autumn)	0.166	0.151	-0.130	0.461
Land-use (Urban)	0.161	0.156	-0.146	0.468
Land-use (Rural Wooded)	-0.016	0.163	-0.336	0.303
Land-use (Rural Open)	-0.159	0.159	-0.471	0.152
Land-use (Urban) : Season (Autumn)	-0.422	0.220	-0.854	0.010
**Land-use (Rural Wooded) : Season (Autumn)**	**-0.467**	**0.221**	**-0.901**	**-0.034**
Land-use (Rural Open) : Season (Autumn)	-0.404	0.227	-0.848	0.041

SEASON: SPRING / LAND-USE: RURAL OPEN				
Parameters	Estimate	Std. Error	95% CIs	
			Lower	Upper
(Intercept)	1.856	0.133	1.595	2.117
Apiary Size	-0.055	0.061	-0.174	0.064
Season (Autumn)	0.105	0.101	-0.093	0.302
Land-use (Suburban)	0.159	0.159	-0.152	0.470
**Land-use (Urban)**	**0.320**	**0.136**	**0.054**	**0.586**
Land-use (Rural Wooded)	0.143	0.138	-0.127	0.413
Land-use (Suburban) : Season (Autumn)	0.403	0.227	-0.042	0.848
Land-use (Urban) : Season (Autumn)	-0.019	0.226	-0.461	0.424
Land-use (Rural Wooded) : Season (Autumn)	-0.064	0.227	-0.508	0.380

SEASON: SPRING / LAND-USE: RURAL WOODED				
Parameters	Estimate	Std. Error	95% CIs	
			Lower	Upper
(Intercept)	1.914	0.098	1.722	2.106
Apiary Size	-0.055	0.061	-0.174	0.064
Season (Autumn)	0.095	0.107	-0.115	0.306
Land-use (Rural Open)	-0.143	0.138	-0.413	0.127
Land-use (Suburban)	0.016	0.163	-0.303	0.336
Land-use (Urban)	0.177	0.133	-0.083	0.438
Land-use (Rural Open) : Season (Autumn)	0.064	0.227	-0.380	0.508
**Land-use (Suburban) : Season (Autumn)**	**0.467**	**0.221**	**0.034**	**0.901**
Land-use (Urban) : Season (Autumn)	0.045	0.220	-0.386	0.477

c) Colony strength				
LAND-USE: URBAN				
Parameters	Estimate	Std. Error	95% CIs	
			Lower	Upper
(Intercept)	3.742	0.254	3.244	4.239
Land-use (Suburban)	0.345	0.355	-0.351	1.041
**Land-use (Rural Open)**	**-1.101**	**0.349**	**-1.784**	**-0.418**
Land-use (Rural Wooded)	-0.377	0.355	-1.073	0.319
LAND-USE: SUBURBAN				
Parameters	Estimate	Std. Error	95% CIs	
			Lower	Upper
(Intercept)	4.087	0.249	3.600	4.574
Land-use (Urban)	-0.345	0.355	-1.041	0.351
**Land-use (Rural Open)**	**-1.446**	**0.345**	**-2.121**	**-0.771**
**Land-use (Rural Wooded)**	**-0.722**	**0.351**	**-1.411**	**-0.034**
LAND-USE: RURAL OPEN				
Parameters	Estimate	Std. Error	95% CIs	
			Lower	Upper
(Intercept)	2.641	0.239	2.173	3.109
**Land-use (Suburban)**	**1.446**	**0.345**	**0.771**	**2.121**
**Land-use (Urban)**	**1.101**	**0.349**	**0.418**	**1.784**
**Land-use (Rural Wooded)**	**0.724**	**0.345**	**0.048**	**1.399**
LAND-USE: RURAL WOODED				
Parameters	Estimate	Std. Error	95% CIs	
			Lower	Upper
(Intercept)	3.365	0.249	2.878	3.852
**Land-use (Rural Open)**	**-0.724**	**0.345**	**-1.399**	**-0.048**
**Land-use (Suburban)**	**0.722**	**0.351**	**0.034**	**1.411**
Land-use (Urban)	0.377	0.355	-0.319	1.073

d) *Varroa*				
SEASON: AUTUMN / LAND-USE: URBAN				
Parameters	Estimate	Std. Error	95% CIs	
			Lower	Upper
Count process				
(Intercept)	3.329	0.134	3.067	3.592
**Apiary Size**	**-0.254**	**0.040**	**-0.331**	**-0.177**
**Colony Strength**	**0.106**	**0.025**	**0.057**	**0.155**
**Season (Spring)**	**-2.227**	**0.266**	**-2.750**	**-1.705**
**Land-use (Suburban)**	**-0.604**	**0.095**	**-0.790**	**-0.417**
Land-use (Rural Open)	-0.124	0.088	-0.297	0.049
Land-use (Rural Wooded)	0.115	0.079	-0.039	0.268
**Land-use (Suburban) : Season (Spring)**	**1.445**	**0.324**	**0.810**	**2.079**
**Land-use (Rural Open) : Season (Spring)**	**1.247**	**0.299**	**0.661**	**1.833**
**Land-use (Rural Wooded) : Season (Spring)**	**0.828**	**0.294**	**0.252**	**1.404**
Binomial process				
(Intercept)	2.128	0.473	1.201	3.055
**Season (Spring)**	**-1.757**	**0.555**	**-2.845**	**-0.669**
SEASON: AUTUMN / LAND-USE: SUBURBAN				
Parameters	Estimate	Std. Error	95% CIs	
			Lower	Upper
Count process				
(Intercept)	2.725	0.149	2.433	3.017
**Apiary Size**	**-0.254**	**0.040**	**-0.331**	**-0.177**
**Colony Strength**	**0.106**	**0.025**	**0.057**	**0.155**
**Season (Spring)**	**-0.783**	**0.183**	**-1.142**	**-0.424**
**Land-use (Urban)**	**0.604**	**0.095**	**0.417**	**0.790**
**Land-use (Rural Open)**	**0.480**	**0.104**	**0.275**	**0.685**
**Land-use (Rural Wooded)**	**0.718**	**0.096**	**0.530**	**0.907**
**Land-use (Urban) : Season (Spring)**	**-1.445**	**0.324**	**-2.079**	**-0.810**
Land-use (Rural Open) : Season (Spring)	-0.198	0.230	-0.649	0.254
**Land-use (Rural Wooded) : Season (Spring)**	**-0.617**	**0.222**	**-1.051**	**-0.183**
Binomial process				
(Intercept)	2.128	0.473	1.201	3.055
**Season (Spring)**	**-1.757**	**0.555**	**-2.845**	**-0.669**
SEASON: AUTUMN / LAND-USE: RURAL OPEN				
Parameters	Estimate	Std. Error	95% CIs	
			Lower	Upper
Count process				
(Intercept)	3.205	0.134	2.943	3.467
**Apiary Size**	**-0.254**	**0.040**	**-0.331**	**-0.177**
**Colony Strength**	**0.106**	**0.025**	**0.057**	**0.155**
**Season (Spring)**	**-0.981**	**0.138**	**-1.251**	**-0.710**
**Land-use (Suburban)**	**-0.480**	**0.104**	**-0.685**	**-0.275**
Land-use (Urban)	0.124	0.088	-0.049	0.297
**Land-use (Rural Wooded)**	**0.238**	**0.086**	**0.070**	**0.407**
Land-use (Suburban) : Season (Spring)	0.198	0.230	-0.254	0.649
**Land-use (Urban) : Season (Spring)**	**-1.247**	**0.299**	**-1.833**	**-0.661**
**Land-use (Rural Wooded) : Season (Spring)**	**-0.419**	**0.186**	**-0.783**	**-0.055**
Binomial process				
(Intercept)	2.128	0.473	1.201	3.055
**Season (Spring)**	**-1.757**	**0.555**	**-2.845**	**-0.669**

SEASON: AUTUMN / LAND-USE: RURAL WOODED				
Parameters	Estimate	Std. Error	95% CIs	
			Lower	Upper
Count process				
(Intercept)	3.444	0.131	3.188	3.700
**Apiary Size**	**-0.254**	**0.040**	**-0.331**	**-0.177**
**Colony Strength**	**0.106**	**0.025**	**0.057**	**0.155**
**Season (Spring)**	**-1.400**	**0.124**	**-1.643**	**-1.156**
**Land-use (Rural Open)**	**-0.238**	**0.086**	**-0.407**	**-0.070**
**Land-use (Suburban)**	**-0.718**	**0.096**	**-0.907**	**-0.530**
Land-use (Urban)	-0.115	0.079	-0.268	0.039
**Land-use (Rural Open) : Season (Spring)**	**0.419**	**0.186**	**0.055**	**0.783**
**Land-use (Suburban) : Season (Spring)**	**0.617**	**0.222**	**0.183**	**1.051**
**Land-use (Urban) : Season (Spring)**	**-0.828**	**0.294**	**-1.404**	**-0.252**
Binomial process				
(Intercept)	2.128	0.473	1.201	3.055
**Season (Spring)**	**-1.757**	**0.555**	**-2.845**	**-0.669**

SEASON: SPRING / LAND-USE: URBAN				
Parameters	Estimate	Std. Error	95% CIs	
			Lower	Upper
Count process				
(Intercept)	1.102	0.282	0.550	1.654
**Apiary Size**	**-0.254**	**0.040**	**-0.331**	**-0.177**
**Colony Strength**	**0.106**	**0.025**	**0.057**	**0.155**
**Season (Autumn)**	**2.227**	**0.266**	**1.705**	**2.750**
**Land-use (Rural Wooded)**	**0.942**	**0.283**	**0.387**	**1.497**
**Land-use (Rural Open)**	**1.123**	**0.288**	**0.559**	**1.687**
**Land-use (Suburban)**	**0.841**	**0.310**	**0.233**	**1.448**
**Land-use (Rural Wooded) : Season (Autumn)**	**-0.828**	**0.294**	**-1.404**	**-0.252**
**Land-use (Rural Open) : Season (Autumn)**	**-1.247**	**0.299**	**-1.833**	**-0.661**
**Land-use (Suburban) : Season (Autumn)**	**-1.445**	**0.324**	**-2.079**	**-0.810**
Binomial process				
(Intercept)	0.372	0.291	-0.198	0.941
**Season (Autumn)**	**1.757**	**0.555**	**0.669**	**2.845**

SEASON: SPRING / LAND-USE: SUBURBAN				
Parameters	Estimate	Std. Error	95% CIs	
			Lower	Upper
Count process				
(Intercept)	1.942	0.215	1.521	2.364
**Apiary Size**	**-0.254**	**0.040**	**-0.331**	**-0.177**
**Colony Strength**	**0.106**	**0.025**	**0.057**	**0.155**
**Season (Autumn)**	**0.783**	**0.183**	**0.424**	**1.142**
**Land-use (Urban)**	**-0.841**	**0.310**	**-1.448**	**-0.233**
Land-use (Rural Wooded)	0.101	0.201	-0.292	0.495
Land-use (Rural Open)	0.282	0.210	-0.129	0.693
**Land-use (Urban) : Season (Autumn)**	**1.445**	**0.324**	**0.810**	**2.079**
**Land-use (Rural Wooded) : Season (Autumn)**	**0.617**	**0.222**	**0.183**	**1.051**
Land-use (Rural Open) : Season (Autumn)	0.198	0.230	-0.254	0.649
Binomial process				
(Intercept)	0.372	0.291	-0.198	0.941
**Season (Autumn)**	**1.757**	**0.555**	**0.669**	**2.845**

SEASON: SPRING / LAND-USE: RURAL OPEN				
Parameters	Estimate	Std. Error	95% CIs	
			Lower	Upper
Count process				
(Intercept)	2.225	0.153	1.925	2.524
**Apiary Size**	**-0.254**	**0.040**	**-0.331**	**-0.177**
**Colony Strength**	**0.106**	**0.025**	**0.057**	**0.155**
**Season (Autumn)**	**0.981**	**0.138**	**0.710**	**1.251**
Land-use (Suburban)	-0.282	0.210	-0.693	0.129
**Land-use (Urban)**	**-1.123**	**0.288**	**-1.687**	**-0.559**
Land-use (Rural Wooded)	-0.181	0.166	-0.506	0.144
Land-use (Suburban) : Season (Autumn)	-0.198	0.230	-0.649	0.254
**Land-use (Urban) : Season (Autumn)**	**1.247**	**0.299**	**0.661**	**1.833**
**Land-use (Rural Wooded) : Season (Autumn)**	**0.419**	**0.186**	**0.055**	**0.783**
Binomial process				
(Intercept)	0.372	0.291	-0.198	0.941
**Season (Autumn)**	**1.757**	**0.555**	**0.669**	**2.845**

SEASON: SPRING / LAND-USE: RURAL WOODED				
Parameters	Estimate	Std. Error	95% CIs	
			Lower	Upper
Count process				
(Intercept)	2.044	0.159	1.733	2.355
**Apiary Size**	**-0.254**	**0.040**	**-0.331**	**-0.177**
**Colony Strength**	**0.106**	**0.025**	**0.057**	**0.155**
**Season (Autumn)**	**1.400**	**0.124**	**1.156**	**1.643**
Land-use (Rural Open)	0.181	0.166	-0.144	0.506
Land-use (Suburban)	-0.101	0.201	-0.495	0.292
**Land-use (Urban)**	**-0.942**	**0.283**	**-1.497**	**-0.387**
**Land-use (Rural Open) : Season (Autumn)**	**-0.419**	**0.186**	**-0.783**	**-0.055**
**Land-use (Suburban) : Season (Autumn)**	**-0.617**	**0.222**	**-1.051**	**-0.183**
**Land-use (Urban) : Season (Autumn)**	**0.828**	**0.294**	**0.252**	**1.404**
Binomial process				
(Intercept)	0.372	0.291	-0.198	0.941
**Season (Autumn)**	**1.757**	**0.555**	**0.669**	**2.845**

e) *Nosema*				
SEASON: AUTUMN / LAND-USE: URBAN				
Parameters	Estimate	Std. Error	95% CIs	
			Lower	Upper
Count process				
(Intercept)	4.495	0.056	4.386	4.605
Apiary Size	-0.009	0.018	-0.044	0.027
Season (Spring)	0.073	0.068	-0.059	0.206
Land-use (Suburban)	0.110	0.065	-0.018	0.238
**Land-use (Rural Open)**	**0.397**	**0.062**	**0.275**	**0.519**
**Land-use (Rural Wooded)**	**0.310**	**0.063**	**0.186**	**0.433**
**Land-use (Suburban) : Season (Spring)**	**-0.299**	**0.089**	**-0.474**	**-0.125**
**Land-use (Rural Open) : Season (Spring)**	**-0.369**	**0.085**	**-0.537**	**-0.202**
**Land-use (Rural Wooded) : Season (Spring)**	**-0.414**	**0.086**	**-0.583**	**-0.246**
Binomial process				
(Intercept)	-0.241	0.482	-1.186	0.705
Apiary Size	0.431	0.279	-0.116	0.978
SEASON: AUTUMN / LAND-USE: SUBURBAN				
Parameters	Estimate	Std. Error	95% CIs	
			Lower	Upper
Count process				
(Intercept)	4.605	0.040	4.527	4.683
Apiary Size	-0.009	0.018	-0.044	0.027
**Season (Spring)**	**-0.226**	**0.057**	**-0.338**	**-0.114**
Land-use (Urban)	-0.110	0.065	-0.238	0.018
**Land-use (Rural Open)**	**0.287**	**0.051**	**0.188**	**0.386**
**Land-use (Rural Wooded)**	**0.200**	**0.051**	**0.100**	**0.300**
**Land-use (Urban) : Season (Spring)**	**0.299**	**0.089**	**0.125**	**0.474**
Land-use (Rural Open) : Season (Spring)	-0.070	0.078	-0.223	0.083
Land-use (Rural Wooded) : Season (Spring)	-0.115	0.078	-0.268	0.038
Binomial process				
(Intercept)	-0.241	0.482	-1.186	0.705
Apiary Size	0.431	0.279	-0.116	0.978
SEASON: AUTUMN / LAND-USE: RURAL OPEN				
Parameters	Estimate	Std. Error	95% CIs	
			Lower	Upper
Count process				
(Intercept)	4.892	0.038	4.818	4.967
Apiary Size	-0.009	0.018	-0.044	0.027
**Season (Spring)**	**-0.296**	**0.053**	**-0.399**	**-0.192**
Land-use (Rural Wooded)	-0.087	0.048	-0.180	0.006
**Land-use (Suburban)**	**-0.287**	**0.051**	**-0.386**	**-0.188**
**Land-use (Urban)**	**-0.397**	**0.062**	**-0.519**	**-0.275**
Land-use (Rural Wooded) : Season (Spring)	-0.045	0.075	-0.192	0.102
Land-use (Suburban) : Season (Spring)	0.070	0.078	-0.083	0.223
**Land-use (Urban) : Season (Spring)**	**0.369**	**0.085**	**0.202**	**0.537**
Binomial process				
(Intercept)	-0.241	0.482	-1.186	0.705
Apiary Size	0.431	0.279	-0.116	0.978

SEASON: AUTUMN / LAND-USE: RURAL WOODED				
Parameters	Estimate	Std. Error	95% CIs	
			Lower	Upper
Count process				
(Intercept)	4.805	0.038	4.731	4.879
Apiary Size	-0.009	0.018	-0.044	0.027
**Season (Spring)**	**-0.341**	**0.053**	**-0.445**	**-0.237**
Land-use (Rural Open)	0.087	0.048	-0.006	0.180
**Land-use (Suburban)**	**-0.200**	**0.051**	**-0.300**	**-0.100**
**Land-use (Urban)**	**-0.310**	**0.063**	**-0.433**	**-0.186**
Land-use (Rural Open) : Season (Spring)	0.045	0.075	-0.102	0.192
Land-use (Suburban) : Season (Spring)	0.115	0.078	-0.038	0.268
**Land-use (Urban) : Season (Spring)**	**0.414**	**0.086**	**0.246**	**0.583**
Binomial process				
(Intercept)	-0.241	0.482	-1.186	0.705
Apiary Size	0.431	0.279	-0.116	0.978

SEASON: SPRING / LAND-USE: URBAN				
Parameters	Estimate	Std. Error	95% CIs	
			Lower	Upper
Count process				
(Intercept)	4.569	0.043	4.484	4.654
Apiary Size	-0.009	0.018	-0.044	0.027
Season (Autumn)	-0.073	0.068	-0.206	0.059
Land-use (Rural Wooded)	-0.104	0.058	-0.219	0.010
Land-use (Rural Open)	0.028	0.059	-0.087	0.143
**Land-use (Suburban)**	**-0.190**	**0.060**	**-0.307**	**-0.072**
**Land-use (Rural Wooded) : Season (Autumn)**	**0.414**	**0.086**	**0.246**	**0.583**
**Land-use (Rural Open) : Season (Autumn)**	**0.369**	**0.085**	**0.202**	**0.537**
**Land-use (Suburban) : Season (Autumn)**	**0.299**	**0.089**	**0.125**	**0.474**
Binomial process				
(Intercept)	-0.241	0.482	-1.186	0.705
Apiary Size	0.431	0.279	-0.116	0.978

SEASON: SPRING / LAND-USE: SUBURBAN				
Parameters	Estimate	Std. Error	95% CIs	
			Lower	Upper
Count process				
(Intercept)	4.379	0.046	4.288	4.470
Apiary Size	-0.009	0.018	-0.044	0.027
**Season (Autumn)**	**0.226**	**0.057**	**0.114**	**0.338**
**Land-use (Urban)**	**0.190**	**0.060**	**0.072**	**0.307**
Land-use (Rural Wooded)	0.085	0.059	-0.030	0.201
**Land-use (Rural Open)**	**0.218**	**0.059**	**0.102**	**0.334**
**Land-use (Urban) : Season (Autumn)**	**-0.299**	**0.089**	**-0.474**	**-0.125**
Land-use (Rural Wooded) : Season (Autumn)	0.115	0.078	-0.038	0.268
Land-use (Rural Open) : Season (Autumn)	0.070	0.078	-0.083	0.223
Binomial process				
(Intercept)	-0.241	0.482	-1.186	0.705
Apiary Size	0.431	0.279	-0.116	0.978

SEASON: SPRING / LAND-USE: RURAL OPEN				
Parameters	Estimate	Std. Error	95% CIs	
			Lower	Upper
Count process				
(Intercept)	4.597	0.044	4.511	4.682
Apiary Size	-0.009	0.018	-0.044	0.027
**Season (Autumn)**	**0.296**	**0.053**	**0.192**	**0.399**
**Land-use (Rural Wooded)**	**-0.132**	**0.058**	**-0.246**	**-0.019**
**Land-use (Suburban)**	**-0.218**	**0.059**	**-0.334**	**-0.102**
Land-use (Urban)	-0.028	0.059	-0.143	0.087
Land-use (Rural Wooded) : Season (Autumn)	0.045	0.075	-0.102	0.192
Land-use (Suburban) : Season (Autumn)	-0.070	0.078	-0.223	0.083
**Land-use (Urban) : Season (Autumn)**	**-0.369**	**0.085**	**-0.537**	**-0.202**
Binomial process				
(Intercept)	-0.241	0.482	-1.186	0.705
Apiary Size	0.431	0.279	-0.116	0.978

SEASON: SPRING / LAND-USE: RURAL WOODED				
Parameters	Estimate	Std. Error	95% CIs	
			Lower	Upper
Count process				
(Intercept)	4.464	0.043	4.379	4.550
Apiary Size	-0.009	0.018	-0.044	0.027
**Season (Autumn)**	**0.341**	**0.053**	**0.237**	**0.445**
**Land-use (Rural Open)**	**0.132**	**0.058**	**0.019**	**0.246**
Land-use (Suburban)	-0.085	0.059	-0.201	0.030
Land-use (Urban)	0.104	0.058	-0.010	0.219
Land-use (Rural Open) : Season (Autumn)	-0.045	0.075	-0.192	0.102
Land-use (Suburban) : Season (Autumn)	-0.115	0.078	-0.268	0.038
**Land-use (Urban) : Season (Autumn)**	**-0.414**	**0.086**	**-0.583**	**-0.246**
Binomial process				
(Intercept)	-0.241	0.482	-1.186	0.705
Apiary Size	0.431	0.279	-0.116	0.978
